# Putative functional genes in idiopathic dilated cardiomyopathy

**DOI:** 10.1038/s41598-017-18524-2

**Published:** 2018-01-08

**Authors:** Nishanth Ulhas Nair, Avinash Das, Uri Amit, Welles Robinson, Seung Gu Park, Mahashweta Basu, Alex Lugo, Jonathan Leor, Eytan Ruppin, Sridhar Hannenhalli

**Affiliations:** 1 Center for Bioinformatics and Computational Biology, University of Maryland, College Park, Maryland 20742 USA; 2 The Neufeld Cardiac Research Institute, Tel Aviv University, Tel Aviv-Yafo, Israel; 3 Tamman Cardiovascular Research Institute, Sheba Medical Center, Ramat Gan, Israel; 4 The Dr. Pinchas Borenstein Talpiot Medical Leadership Program, Sheba Medical Center, Tel-Hashomer, Israel; 5 Department of Radiation Oncology, Sheba Medical Center, Tel-Hashomer, Israel; 6 The Blavatnik School of Computer Science, Tel Aviv University, Tel Aviv, 69978 Israel

## Abstract

Idiopathic dilated cardiomyopathy (DCM) is a complex disorder with a genetic and an environmental component involving multiple genes, many of which are yet to be discovered. We integrate genetic, epigenetic, transcriptomic, phenotypic, and evolutionary features into a method – *Hridaya*, to infer putative functional genes underlying DCM in a genome-wide fashion, using 213 human heart genomes and transcriptomes. Many genes identified by Hridaya are experimentally shown to cause cardiac complications. We validate the top predicted genes, via five different genome-wide analyses: First, the predicted genes are associated with cardiovascular functions. Second, their knockdowns in mice induce cardiac abnormalities. Third, their inhibition by drugs cause cardiac side effects in human. Fourth, they tend to have differential exon usage between DCM and normal samples. Fifth, analyzing 213 individual genotypes, we show that regulatory polymorphisms of the predicted genes are associated with elevated risk of cardiomyopathy. The stratification of DCM patients based on cardiac expression of the functional genes reveals two subgroups differing in key cardiac phenotypes. Integrating predicted functional genes with cardiomyocyte drug treatment experiments reveals novel potential drug targets. We provide a list of investigational drugs that target the newly identified functional genes that may lead to cardiac side effects.

## Introduction

Heart failure is a major cause of morbidity and mortality worldwide^1^. A major cause of heart failure is cardiomyopathy, which is a disease of the heart muscle where the heart muscle becomes enlarged, rigid, or thick^2^. Idiopathic dilated cardiomyopathy (DCM) is one of the most common forms of cardiomyopathy and is characterized by enlarged and weakened ventricles^2^, resulting in reduced ability of the heart to pump blood. DCM is a complex disorder caused by the dysregulation of multiple genes and is shown to have genetic basis^3^. However, the molecular mechanisms and the functional genes underlying DCM remain poorly understood, and present a critical bottleneck in early diagnosis and the design of rational DCM therapies. Here the term ‘functional’ is used to refer to the genes that are involved in processes and pathways whose disruption is functionally linked to DCM.

Differential gene expression analysis has previously been used to identify key genes involved in DCM^4–8^. However, functional genes may not have a globally detectable differential expression (as they may have aberrant expression in only a subset of DCM individuals), and conversely, differential expression alone cannot distinguish functional genes from the downstream effects or co-expressed genes. Thus, additional genetic, epigenetic, and evolutionary features need to be considered. For instance, a recent paper^9^ inferred gene co-expression network using diseased and healthy hearts and prioritized genes based on the differential network topology. They then identified PPP1R3A as a critical gene and experimentally verified its association with heart failure. Various studies have also used animal models to test the role of specific genes in cardiomyopathy^10^. However, genes predicted using animal models do not always translate to humans. Importantly, the previous studies have not considered the genetic signals underlying the differentially expressed genes and the association of those signals with DCM to detect potentially functional genes.

Here we integrate genetic, epigenetic, transcriptomic, phenotypic, and evolutionary features, 181 features in total, in a machine-learning method, called *Hridaya*, to identify potential human DCM functional genes in a genome-wide fashion. In a two-step supervised training, Hridaya estimates the (1) potential of a gene to be a functional gene of any disease in general, and (2) specifically, the potential of any disease functional gene to be a functional gene of DCM; these two estimates are combined to obtain the potential for a gene to be a function gene of DCM. We train Hridaya using genetic, transcriptomic, and phenotypic data from 77 DCM human hearts and 136 human hearts from donor controls from the Myocardial Applied Genomics Network (MAGNet, http://www.med.upenn.edu/magnet/, GSE57338) cohort, as well as a variety of additional transcriptomic, epigenomic, and phylogenetic data from public resources^11–18^. We show that the top Hridaya-predicted functional genes (called *Hridaya-genes*) exhibit cardiomyopathy-related phenotypes in mouse knockout data and that commonly used drugs that target Hridaya-genes tend to have cardiac side effects. Hridaya-genes are also shown to have differential exon usage between DCM and normal patients in an independent dataset. Importantly, the SNPs (single nucleotide polymorphisms) that are linked with the population variance of the expression of Hridaya-genes directly associate with a higher occurrence of cardiomyopathy, suggesting transcriptional dysregulation of the Hridaya-genes in an individual are likely to underlie cardiomyopathies.

We find that the Hridaya-genes, many of which were previously not known to be involved in heart diseases, nevertheless show clear associations with cardiovascular functions. Furthermore, our analysis suggests that Hridaya-genes are upstream regulators of genes that are differentially expressed in failing hearts. Finally, stratification of DCM patients, based on functional gene expression, or based on associated eQTL (expression quantitative trait loci) SNPs, revealed two distinct subgroups of DCM patients having significant differences in multiple phenotypes, notably, left ventricular end-systolic/diastolic diameters. Integrating data from drug treatment experiments on cardiomyocytes and the Hridaya-genes, we identify potential novel drug targets for DCM. To aid future targeted investigations, we provide a list of a few investigational drugs that target the newly predicted DCM functional genes and thus may have an increased likelihood of cardiac side effects. We also developed a web-application which may be useful for biologists and bioinformaticians to further explore functional genes of their interest.

## Results

### Hridaya overview

**Figure 1:**
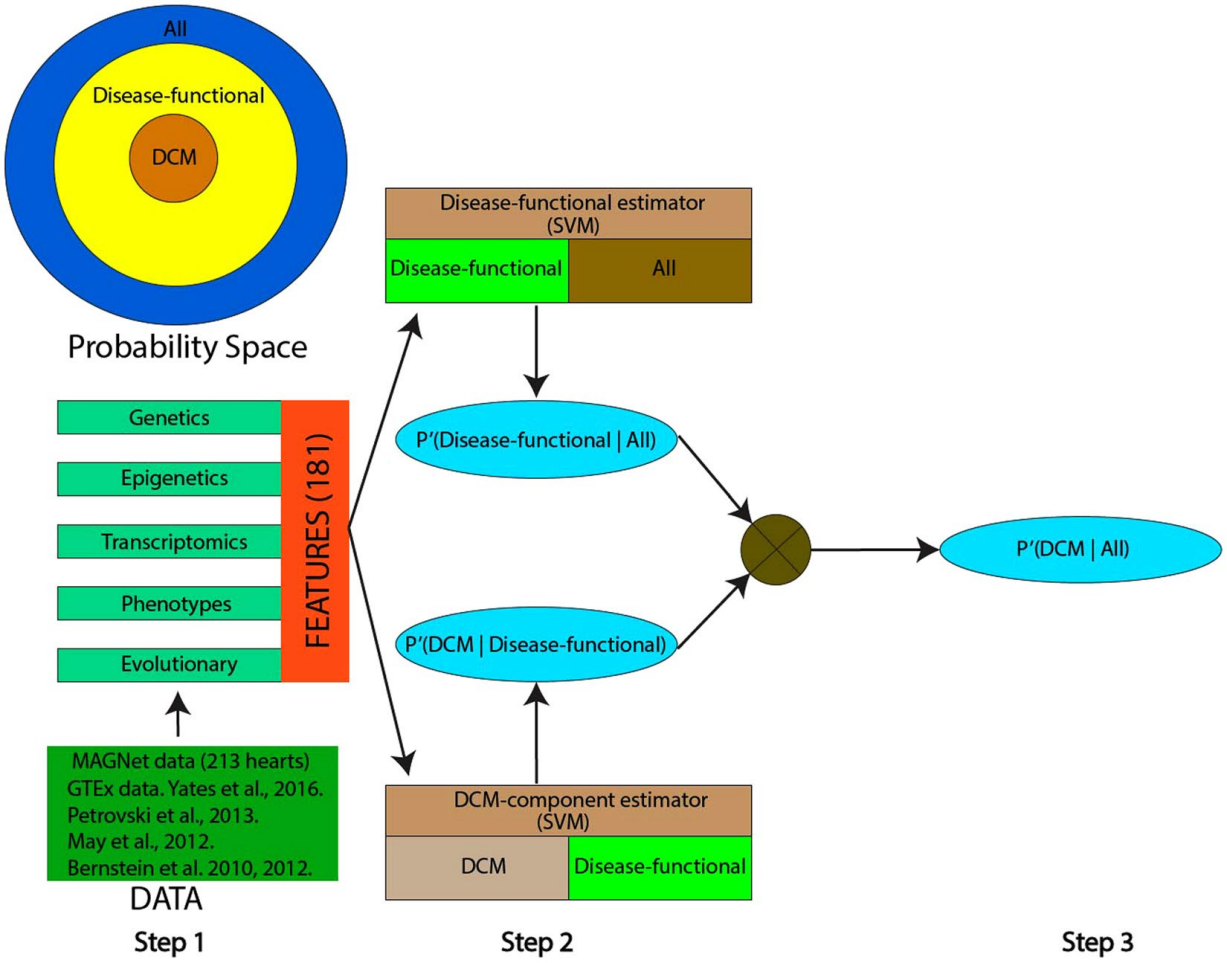
Outline of the Hridaya method to predict functional genes for DCM. Each gene is represented by genetic, epigenetic, transcriptomic, phenotypic, and evolutionary features. The disease-functional estimator predicts the probability of a gene to be a functional gene of any disease. The DCM-component estimator estimates the probability of a disease-functional gene to be functional gene of DCM. The two probabilities are multiplied to get the probability of a gene being a functional gene of DCM. The probability space of disease-functional gene and a functional gene of DCM is shown in top left.

Integrating genetic, epigenetic, transcriptomic, phenotypic, network, and evolutionary features (Supplementary Table S1), Hridaya hierarchically estimates the potential of a gene to be a functional gene of DCM in a supervised fashion (Fig. 1). Hridaya comprises of two supervised Support Vector Machines (SVM) based models, *Disease-functional estimator*, and *DCM-component estimator* (see Methods). The Disease-functional estimator estimates the probability of a gene to be a functional gene of any disease (called disease-functional gene) by training on 3373 known disease associated genes in the Human Phenotype Ontology (HPO) dataset^19^. The Disease-functional estimator model is trained to discriminate between two classes of genes, those that are known to be functionally linked to any human disease in HPO, against the rest of the genes. The Disease-functional estimator estimates the probability of a gene to be functionally linked to a human disease. Similarly, the DCM-component estimator model is trained to discriminate between two classes of genes, those that are functionally linked to DCM (214 gold-standard gene set; Supplementary Table S2(b)) and the set of all other disease-linked genes. The DCM-component estimator estimates the probability of a disease-functional gene to be linked specifically to DCM. Finally, we test all genes using both the SVMs, and for each gene we multiply the probabilities estimated by the two components to estimate the overall potential (called Hridaya-potential) of a gene to be functionally linked to DCM (DCM functional gene).

Hridaya uses Support Vector Machines (SVM) for both models and represents genes using 181 features from genetic, epigenetic, transcriptomic, phenotypic, and evolutionary data. A representative list of features is shown in Table 1 (Supplementary Table S1 provides the full list). We evaluate the five-fold prediction accuracy (with 50 iterations) of the model using standard *Receiver Operating Characteristics Area Under the Curve (ROC-AUC)*. Finally, we train Hridaya on the entire gold-standard set of genes and rank all human protein coding genes by their predicted Hridaya-potential, which is provided in Supplementary Table S2(a,d).

**Table 1:** Representative features used to train Hridaya.

Feature type	Representative features
Transcriptomic	• Differential gene expression (and its absolute value) between DCM and normal individual heart samples.• Median gene expression of DCM and normal individuals (MAGNet, http://www.med.upenn.edu/magnet/, GSE57338).• The number of tissues, based on GTEx data^13,14^, for which gene expression is significantly more (or less) than gene expression of the gene in the left ventricle (LV) and LV-tissue specific expression rank (LV-tissue specific features).
Transcriptomic and Phenotypic	• Correlation of gene expression with various phenotypes of the individuals in MAGNet data.
Genetic	• GWAS (Genome-wide association studies) signal of eQTL SNPs of the gene.
Evolutionary	• Phylogenetic profile of the gene with 65 species^17^.• Residual variation intolerance score (RVIS)^16^.• D_N_/D_S_ ratio between human and mouse^17^.
Epigenetic	• Signals of various histone marks, transcription factors from Left/Right Ventricle, Aorta, Fetal Heart^11,12^.• p300 transcription factor signal (GSM807734^15^) around transcription start site of the gene from the adult heart (p300 is often seen as an enhancer mark^68,69^).• DNase hypersensitivity data around the gene transcription start site^11,12^.

The detailed list of 181 features are provided in Supplementary Table S1.

### Hridaya accurately predicts the known functional genes in human

**Figure 2:**
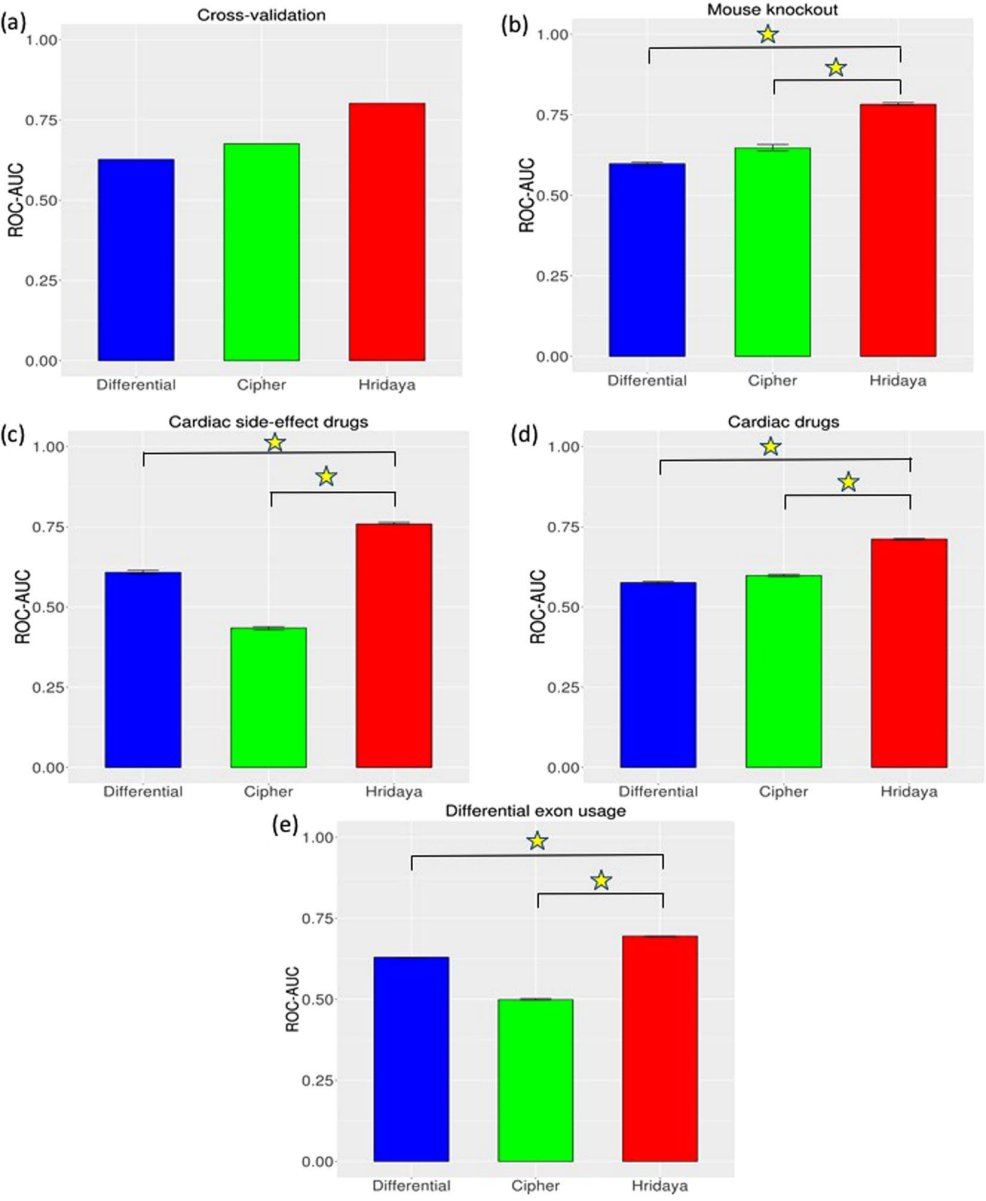
Comparison of Hridaya method with Cipher, and conventional differential gene expression predictor (Differential), based on (**a**) Cross-validation in known DCM genes in HPO database, (**b**) DCM related mouse knockout genes, (**c**) targets of drugs administered for cardiac phenotypes, (**d**) targets of drugs that have cardiac side-effects, (**e**) genes having significant differential exon usage in DCM. Confidence intervals are based on 100 bootstrap samplings. Yellow star indicates a significant p-value < 2.2e-16 (using Wilcoxon test) between the accuracies of the methods.

Hridaya accurately predicts the known DCM-linked functional genes listed in the HPO dataset (five-fold cross-validation ROC-AUC = 0.805, see Methods; precision-recall AUC = 0.245, see supplementary note). Its accuracy is much higher (Wilcoxon test p-value < 2.2 E-16; Methods; Fig. 2a) compared to the accuracy of a conventional differential expression based predictor (ROC-AUC = 0.627), which ranks genes based simply on the differential expression between DCM and normal individuals (see Methods). We also compare Hridaya with another method called Cipher^20^ that identifies disease causing genes using protein-protein interaction networks (Methods). Cipher predicts the known DCM-linked genes with an ROC-AUC of 0.676.

Among the top 1000 Hridaya-genes (Supplementary Table S2(a)) 64.27% were downregulated in DCM individual while 19.56% were upregulated in DCM individuals, relative to control. We compare the individual predictive power of each feature and find that the evolutionary features, especially the phylogenetic profile of a gene, are the strongest predictors for the Disease-functional estimator. While for the DCM-component estimator, the most predictive features were median gene expression in DCM and normal individuals, LV-tissue specific features, and epigenetic features (see Methods). ROC-AUC values using one feature at a time are also computed using the entire Hridaya pipeline (Supplementary Table S1, see Methods). As a check, we see that the box plot of ROC-AUCs on cross-validation results for Hridaya using all features (Supplementary Fig. S1) show that our predictions are very stable.

We included evolutionary features because conservation is indicative of functionality. However, there is a concern that more conserved genes are more likely to be explored and therefore functionally annotated. To ensure that a differential conservation between the positive and the negative sets does not bias our prediction accuracy, we repeated the five-fold cross-validation analysis using two alternatives: (1) We only included genes (in both the positive and the negative sets) that have an ortholog in at least one of the 65 species used. We obtained an ROC-AUC of 0.803, almost same as the accuracy obtained without this filtering step (ROC-AUC = 0.805). (2) We included only the genes (in both the positive and the negative sets) that have an ortholog in mouse and repeated the analysis. Again, we obtained an ROC-AUC of 0.782. Thus, the suspected conservation bias does not affect our results.

To check if our results are biased due to various confounders, we used Limma R package^21^ to remove the contribution of potential confounding factors such age, sex, ethnicity, race, BMI, history of hypertension, etc. from the overall gene expression. We then re-trained the Hridaya method using the corrected gene expression. We find a Spearman correlation of 0.91 (p-value < 2.2e-16) between the Hridaya potentials using this approach from what we found earlier (prior to this correction), suggesting that the Hridaya potentials are not biased by the potential confounders.

### Hridaya accurately predicts the known functional genes in mouse

Next, using gene-knockout information from the mouse knockout database^22^, we tested whether the knockout of the mouse ortholog of a Hridaya-gene results in abnormal cardiac phenotypes in mice. Specifically, we compiled 34 genes whose knockout results in DCM-related phenotypes (Supplementary Table S2(c)), and find that Hridaya accurately identified these genes (ROC-AUC = 0.783, Fig. 2b), which was significantly higher (p-value < 2.2e-16) than a conventional differential gene expression predictor (ROC-AUC = 0.598) and Cipher (ROC-AUC = 0.647). The Hridaya-potentials for the genes identified by the mouse-knockout experiments were significantly greater than the rest of the genes with p-value = 2.54e-08 (one-sided Wilcoxon rank-sum test). For instance, knockout of top Hridaya-predicted functional gene MYBPC3 causes increased heart-weight to body weight in mice, an important feature of cardiomyopathy. Interestingly, knockout of Hridaya-predicted genes SLC25A4 and NDUFS3 also caused increased heart weight^22^ and cardiomyopathy in mice (http://www.mousephenotype.org), but are not part of our gold-standard set of known DCM-linked genes. Further, SLC25A4 gene is also linked to autosomal dominant and autosomal recessive cardiomyopathic types of mitochondrial DNA depletion (OMIM database^23^, www.omim.org); which illustrates the power of our integrative approach to identify functional genes underlying DCM.

### Predicted functional genes are enriched for targets of drugs known to be cardiotoxic as well as targets of cardiac drugs

Next, we assessed whether the predicted functional genes are targets of drugs exhibiting cardiomyopathic side effects. We identified 17 such drugs from SIDER drug side-effect database^24,25^, and compiled their 73 targets (Supplementary Table S3(a)) from Drug Bank^26^. Hridaya-potentials accurately predicts these genes with known cardiomyopathic side-effect (ROC-AUC = 0.759), in contrast to ROC-AUC of 0.608 using the conventional differential gene expression predictor and ROC-AUC of 0.435 using Cipher (Fig. 2c). Direct comparison based on bootstrapped accuracy values shows significantly greater accuracy of Hridaya when compared to the conventional differential gene expression predictor and Cipher (p-value < 2.2e-16). Hridaya-potentials for these drug targets were significantly greater than the rest of the genes with p-value 1.17e-13 (one sided Wilcoxon rank-sum test). There are 3 genes that are targets of drugs with cardiomyopathic side effects, which are among the top 1000 Hridaya-genes and are not known DCM-linked genes, and in addition are differentially expressed between DCM-affected and normal individuals. The genes are PDGFRB, ABL1, FLT1; and these genes are drug targets of cancer drugs like Dasatinib (targets – PDGFRB, ABL1), Pazopanib (targets – PDGFRB, FLT1), Ponatinib (target – ABL1)^26^. The known side effects of Dasatinib, Pazopanib and Ponatinib are congestive cardiomyopathy, restrictive cardiomyopathy, and ischaemic cardiomyopathy^24,25^ respectively. Interestingly, we found that all these three genes are downregulated in DCM patients compare to normal subjects, consistent with the inhibitory mode of action of most drugs. This result suggests that the observed cardiac side effects of these drugs may be mediated by the inhibition of these specific predicted functional genes.

We performed an analogous test using the 217 targets of 76 drugs currently used for cardiovascular diseases (Supplementary Table S3(b), Methods). Hridaya-potentials for genes could distinguish the drug targets from the negative control with an ROC-AUC of 0.712, compared with 0.576 using differential gene expression and 0.598 using Cipher (Fig. 2d). A direct comparison showed significant greater accuracy of Hridaya when compared to both differential gene expression based predictor and Cipher (p-value < 2.2e-16). Hridaya-potentials for these drug targets were differentially greater than the rest of the genes with p-value 8.05e-26 (one-sided Wilcoxon rank-sum test). Among the targets of cardiac drugs that were in the top 1000 Hridaya-genes (not including the known DCM-linked genes) and are differentially expressed between DCM and control individuals, we found that 29% (2 out of 7) were upregulated in DCM individuals, compared with none in the cardiac side-effect causing drugs’ targets. The two genes are GUCY1A2 and ACE2 and are targeted by Riociguat and Lisinopril respectively. Riociguat is a stimulator of soluble guanylate cyclase (sGC) and is used treat pulmonary hypertension. Lisinopril is an ACE-inhibitor and is used to treat hypertension and heart failure^26^.

Together, these results suggest that the Hridaya-predicted functional genes may both aid in new drug targets prioritization and identify drugs that may have potential cardiac side effects. As a resource for future clinical investigations, we provide a small list of investigational drugs with Hridaya-predicted cardiac side effects (Supplementary Table S3(c)). Toward this goal we identified investigational drugs from DrugBank^26^ with single known gene target (to minimize ambiguity) and ranked the gene targets using Hridaya-potential. We further filtered this list by keeping only drugs whose targets were significantly under-expressed in heart tissue for DCM individuals compared to normal (since most drugs act as inhibitors). We thus identify 3 investigational drugs, namely Mycobacterial Cell Wall-DNA Complex (MCC), AT2220, and Oprelvekin. MCC has apoptosis and immune stimulatory functions against cancer cells. AT2220 is used to treat Pompe disease^26^. Oprelvekin stimulates platelet production in the blood and is known to cause side effects like fast/irregular heartbeat (www.drugs.com). We also did the same for FDA-approved drugs. Encouragingly, we found that most of the drugs that we predicted to have cardiac side effects where already known to have cardiac side effects. Supplementary Table S3(d) lists the approved drugs that target top Hridaya-genes and their associated cardiac side-effects compiled from the literature survey.

### Predicted functional genes are enriched for genes having differential exon usage in DCM

Alternative splicing has been suggested to play a role in DCM etiology^27–30^. Splicing is often characterized in terms of exon usage^31–33^. We used an independent dataset^30^ that discovered hundreds of genes with differential exon usage between 97 dilated cardiomyopathy patients and 108 non-diseased controls for this analysis. We assessed whether genes having a differential exon usage in DCM are predicted to be functional by our method. Encouragingly, we find that the Hridaya-potentials are much higher for genes having differential exon usage (739 genes) than the rest of the genes (Wilcoxon rank-rum, p-value = 1.31e-73). As an alternative assessment, Hridaya-potentials discriminate the genes that have significant differential exon usage from the rest (ROC-AUC = 0.7), in contrast to ROC-AUC of 0.63 using the conventional differential gene expression predictor and ROC-AUC of 0.5 using Cipher (Fig. 2e); these differences in accuracy between Hridaya and other two methods are both significant (p-values < 2.2e-16).

### Predicted functional genes have potential roles in cardiac function and in regulating differentially expressed genes

We found several examples of high-ranking Hridaya-genes that have not been incorporated in the HPO database (and hence were not used to train the model) but were very recently identified as key players in cardiac function. Notably, the TTL enzyme (Hridaya rank 201 out of more than 20,000 genes) was very recently shown to play an important role in microtubule buckling during cardiac contraction^34^. The overexpression of TTL reduces the density of polymerized microtubule network, which plays various roles in beating cardiomyocyte. Ablation of PPP1R3A gene (Hridaya rank 593) was recently shown to be associated with heart failure^9^. A recent paper showed that transcription factor KLF15 (rank 145) regulates branch chain amino acids (BCAA) catabolism in the heart, which plays an important role in heart failure^35^. Some of the genes in the BCAA metabolic pathway such as MLYCD (rank 164), HADHB (rank 354), IVD (rank 713), MUT (rank 921), and PCCB (rank 684) are also ranked highly by Hridaya. SIRT5 gene (rank 945) was recently identified as an important regulator of cardiac function and is associated with cardiomyopathy in mice^36^. These genes provide potential targets for future investigations. We discuss the potential DCM links of several Hridaya-genes hitherto not linked to cardiomyopathies in Supplementary note.

The widespread differential expression between DCM and healthy hearts suggests substantial secondary regulatory effects originating at functional genes. Analyzing the human functional gene interaction network^37^, we illustrate this secondary effect by testing whether the differentially expressed genes are closer to predicted functional gene than expected in the interaction network. We found that the both mean and minimum path lengths from differential genes to top ranked Hridaya-genes are significantly shorter than those to the bottom ranked genes (p-value < 2.2e-16, Wilcoxon ranked sum test). We validated the robustness of the result by repeating the analysis for different network stringencies (see Methods, Supplementary Fig. S2).

Further, we found that known DCM-linked genes are much closer to the Hridaya-genes compared to other genes (one-sided Wilcoxon rank-sum test p-value < 2.2e-16, for different network stringencies; see Methods and Supplementary note). Note that gene-gene interaction networks were not used in training Hridaya.

To ensure that the above results are not biased by higher co-expression between differential genes and the predicted functional genes, we repeated this analysis after explicitly equalizing the co-expression of the differential and non-differential gene sets relative to the predicted functional genes. We first compute the expression correlation of the top predicted functional genes with differentially expressed genes. Among the negative set of non-differentially expressed genes, we randomly sample genes such that their expression correlation with predicted functional genes have same overall distribution as that of differentially expressed genes. This procedure ensures that the co-expression distributions between the differentially expressed genes and the randomly sampled background set are same with respect to the predicted functional genes. As before, we compute the shortest path of predicted functional genes with respect to the foreground (differentially expressed genes) and selected background. We find that predicted functional genes are much closer to the foreground than the background, as before (Supplementary Fig. S3, Methods). This suggests that differential expression correlation does not bias our results.

Functional enrichment analysis of the top 250 Hridaya-genes (excluding the known DCM genes used for training) based on KEGG pathways and GO biological processes revealed association with many cardiac functions (Supplementary Table S4, supplementary note).

### SNPs linked to expression variance of Hridaya-genes are associated with DCM risk

**Figure 3:**
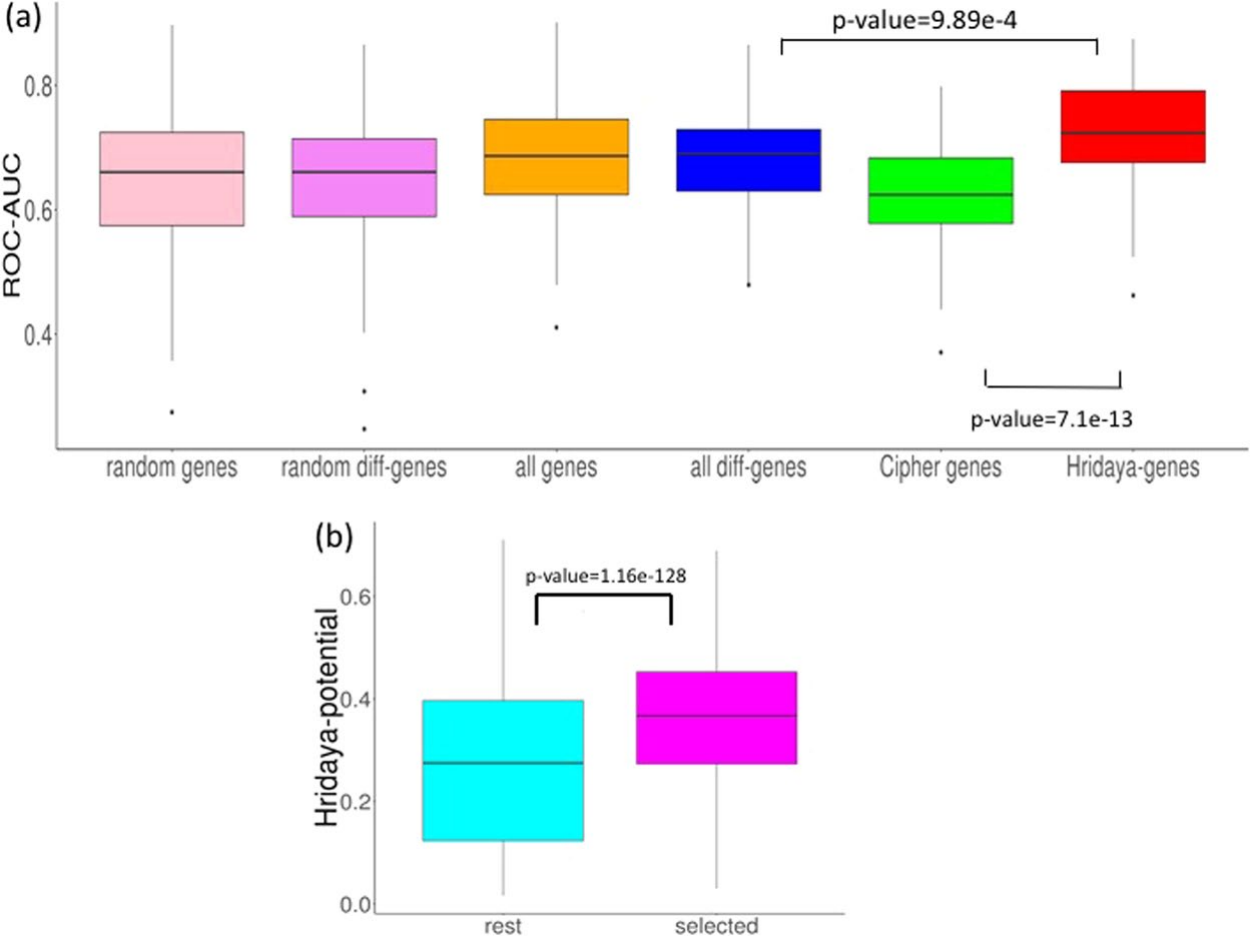
(**a**) Accuracies of predicting DCM individuals based on eSNPs for different gene sets: The gene sets were randomly selected 1000 genes (random genes), randomly selected 1000 genes from differentially expressed genes between DCM and normal patients (random diff-genes), all genes, all differentially expressed genes (all diff-genes), top 1000 Cipher genes, and top 1000 Hridaya-genes. The p-values using Wilcoxon test comparing the accuracies of Hridaya-genes with differential and Cipher genes are shown. (**b**) Cell line experiments: Hridaya-potentials for the selected genes based on cell line experiments (DToxS data) when compared to the rest of the genes. The genes were selected based on their change in expression when treated with a cardiotoxic drug and reversal of gene expression upon treatment with a paired mitigating drug.

DCM is known to have a genetic basis^3^. One mechanism by which our predicted functional genes can affect population variation of DCM is through their expression variation. We hypothesized that, if the predicted Hridaya-genes are functional, polymorphisms associated with their expression should be predictive of DCM incidence in human population. We assessed whether the eQTL SNPs (eSNPs) underlying the population variance of Hridaya-genes’ expression are associated with risk of DCM. Accordingly, using genotypes of 313 individuals, with 8,349,560 SNP genotyped in MAGNet cohort, we first identified the eSNPs, i.e. SNPs associated with expression variance in the human heart (Methods^38^). To avoid circularity, we re-predicted functional genes using Hridaya after removing features related to eSNPs (see Methods). We find eSNPs for a gene based on matrix eQTL method^38^. For each gene, we choose the eSNPs which have an FDR < 0.1, and use their genotypes (with values of 0, 1, or 2) as features (see Methods). We find that the eSNPs of Hridaya-genes accurately predict DCM incidences (Fig. 3a, 5-fold cross validation ROC-AUC of 0.713), which was significantly better than prediction accuracy based on eSNPs of: (a) random-1000 genes (ROC-AUC = 0.66), (b) random-1000 differentially expressed genes (ROC-AUC = 0.66), (c) all differentially expressed genes (ROC-AUC = 0.69), (d) all genes (ROC-AUC = 0.69), or (e) top 1000 disease-genes predicted by Cipher for dilated cardiomyopathy (ROC-AUC = 0.62). These results (Fig. 3a) suggest that predicted functional genes may mediate, in part, the effects of SNPs on DCM.

In addition, we also computed GWAS Chi-square value for eSNPs (if FDR < 0.1) and chose the maximum value for each gene. We find that the GWAS signals for the top 1000 predicted functional genes are significantly higher than those for differentially expressed genes (Wilcoxon, p-value = 3.94e-05).

### Expression changes in cardiomyocytes upon treatment with cardiotoxic and mitigating drugs are consistent with Hridaya and reveal novel candidate drug targets for DCM

Next, we assessed whether transcriptomic changes in cardiomyocytes upon treatment with known cardiotoxic and mitigating drugs are consistent with predicted transcriptomic changes of predicted functional genes. We used the Drug Toxicity Signature (DToxS) Generation Center cell line database (martip03.u.hpc.mssm.edu/data.php), which contains 4 cardiomyocyte cell lines from healthy hearts^39^. Gene expression data (RNA-seq) were obtained in three states: (1) naïve cells, (2) upon treatment with a cardiotoxic (offending) drug, and (3) upon treatment with an offending and a specific known mitigating drug; in total, we obtained 28 triplets of transcriptomic data for cell-line drug pair combinations, Supplementary Table S5(a)). We first identified the genes that are differentially expressed (see Methods) between control and offending drug (in various cell lines) in the same direction as in MAGNet DCM patients compared to normal hearts. Among these genes, we further identify the subset of genes whose expression changes back to the normal state upon treatment with the mitigating drug (see Methods). We find that the Hridaya-potentials for these genes selected from the DToxS data (excluding known DCM-linked genes) are much higher than the remaining genes (p-value = 1.16e-128, Fig. 3b). We also find that these genes are ranked much more highly by Hridaya than by a conventional differential gene expression predictor (Wilcoxon, p-value = 2.33e-08, supplementary Fig. S4, see Methods).

We are particularly interested in genes that are upregulated in cell lines after treatment with the offending drug (and are also upregulated in DCM patients) but are not upregulated after treatment with both the offending and mitigating drugs. Such genes have a potential translational value as a drug target. We identify 33 such genes that are highly ranked by Hridaya and follow the above transcriptomic pattern in multiple cell line drug combinations. Remarkably, 6 genes are already known to be DCM-linked. Among these, ACTC1 gene (Hridaya rank 20) and CASQ2 gene (Hridaya rank 22) exhibit consistent expression changes in 4 cell line drug combinations each. ACTC1 is known to be associated with both DCM and familial hypertrophic cardiomyopathy and is involved in cardiac muscle contraction^40^. CASQ2, which is a cardiac muscle family member of calsequestrin family, is a calcium binding protein that stores calcium for muscle function^40^. EHD3 (Hridaya rank 349, occurs in 4 cell line drug combinations) plays a role in cardiac protein trafficking^41^ and is a component of cardiac remodeling pathway in heart failure^42^. WSF1 (Hridaya rank 474) is known to be associated with cardiomyopathy^43^ and this gene occurs in 8 cell line drug combinations. The list of 33 genes and their details are provided in Supplementary Table S5(b).

### Stratification of DCM patients reveals two subgroups of patients based on their Left Ventricular End Diastolic/Systolic Diameters

**Figure 4:**
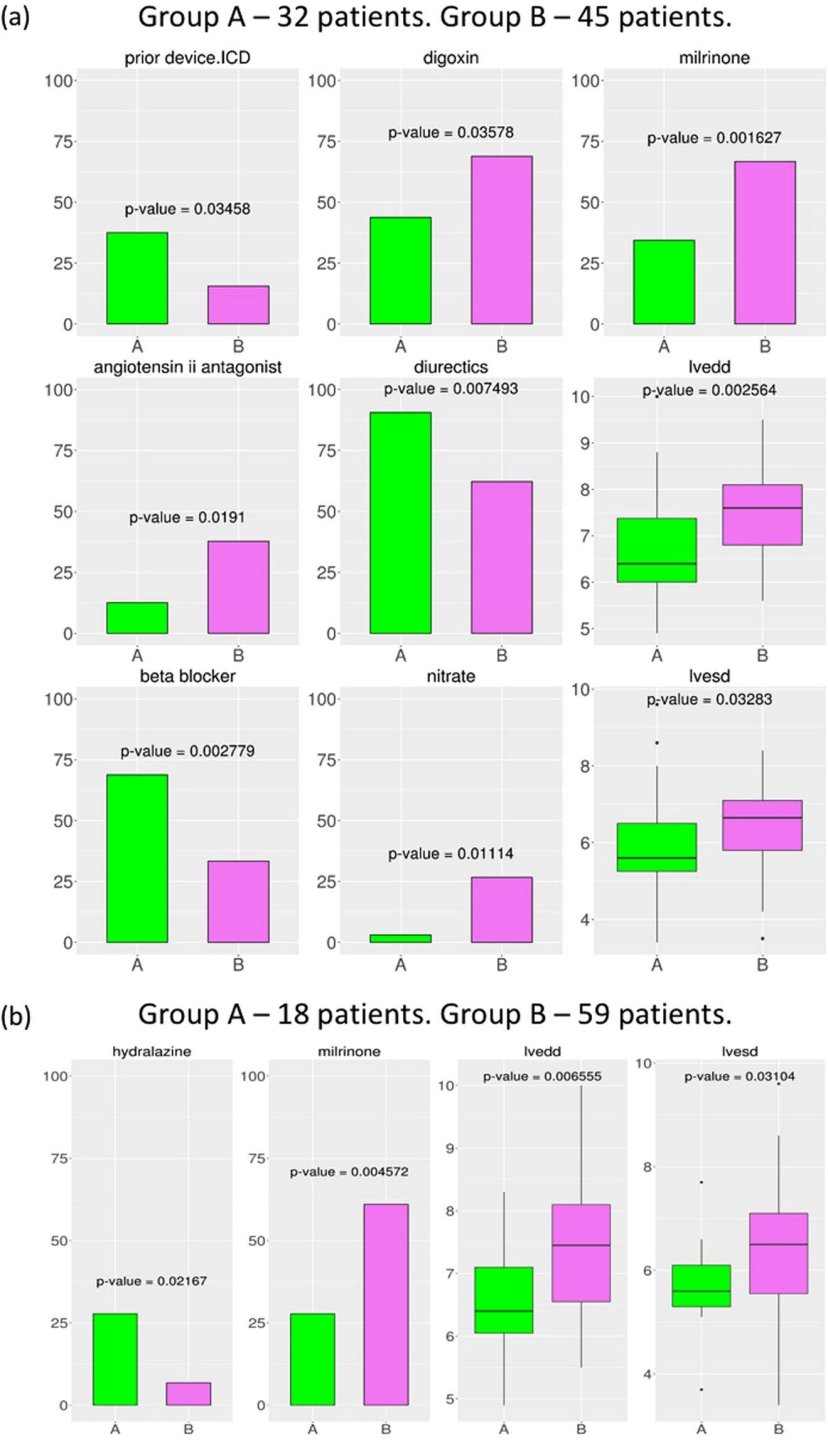
Stratification of DCM patients using transcriptomic and genetic profiles. (**a**) Stratification using gene expression (subgroup A has 32 patients and subgroup B has 45 patients); (**b**) Stratification using eSNPs (subgroup A has 18 patients and subgroup B has 59 patients). 77 DCM patients are stratified based on gene expression or eSNPs of Hridaya-genes. Hierarchical clustering is done, and the patients are divided into two subgroups. The significant phenotypes of these two subgroups are shown. The binary phenotypes are shown as bar plots indicating the percentage (%) of the individuals in each subgroup having a particular phenotype. The continuous valued phenotypes (in centimeter, cm) are shown as box plots. The p-value of the phenotypic differences between the two subgroups using either Wilcoxon rank-sum test (continuous valued phenotype) or Fischer test (binary valued phenotype) are shown in each figure, and the title of each figure shows the phenotype described. Significant phenotypic differences between the two subgroups are observed for LVESD, LVEDD, and if patients have taken drugs like Milronine, Hydralazine, Nitrate, Beta blockers, Angiotensin II Antagonists.

DCM, as a complex and systemic disease, is likely to be molecularly and genetically heterogeneous. That is, different combinations of activity states of the predicted functional genes may represent different etiologies underlying DCM. We therefore explored whether DCM patients form distinct subgroups defined by the Hridaya-genes’ expression profile. Hierarchical clustering of the 77 DCM patients based on the gene expression of the top 1000 Hridaya-genes revealed two subgroups (subgroup A: 32 patients, subgroup B: 45 patients). Each subgroup represents DCM patients with similar heart expression patterns among the predicted functional genes. We analyzed phenotypic differences between the two subgroups for the 59 phenotypes provided in the MAGNet database (Fig. 4a) using the Fisher test^44^ for binary phenotypes and the two-sided Wilcoxon rank-sum test for continuous-valued phenotypes. The two subgroups differed significantly in multiple phenotypes, most notably, the left ventricular end diastolic and systolic diameters (LVEDD, p-value = 2.56e-3; LVESD, p-value = 3.28e-2), which are the key measures of cardiac structural changes and remodeling in heart failure patients (Fig. 4a). These results reveal an association between gene expression profile of the predicted functional genes and key cardiac phenotypes.

Similar to transcriptome-based stratification, next we stratified the DCM patients using the genotypes across the significant eSNPs of the Hridaya-genes (FDR < 0.1). This again yielded two subgroups (subgroup A = 18 patients, subgroup B = 59). (17 out of 18 patients in subgroup A using eSNPs overlap with subgroup A patients using expression profile while 44 out of 45 patients from subgroup B using expression profile overlap with subgroup B patients using eSNPs.) In this SNP-based clustering as well, we found significant differences in LVEDD (p-value = 6.5e-3), LVESD (p-value = 3.1e-2), etc. (Fig. 4b). Consistently, in both stratifications, the subgroup having higher LVEDD and LVESD was more likely to have taken the inotropic drug *Milrinone* ^45^. In expression-based stratification, the other subgroup of patients was more likely to have taken beta-blockers, a class of drugs used to manage chronic heart failure, arrhythmias, and hypertension^46^. These results suggest that patients in subgroup B (who have greater LVEDD and LVESD values), may have a more severe form of DCM, as expected. The consistent results by both SNP-based and expression-based stratification suggest a genetic component underlying the observed transcriptomic and phenotypic heterogeneity among DCM patients.

### Web-application

We have created a web-application (https://nishanthnair.shinyapps.io/heartdisease/) that allows users to search for and view the following information for every gene: the Hridaya rank, the gene description, a link to the GeneCards website (http://www.genecards.org)^40^, the ratio of expression between normal and diseased DCM individuals, etc.

## Discussion

We have reported a novel machine learning approach called *Hridaya* to predict DCM functional genes. Building on the existing knowledge of DCM functional genes, Hridaya attempts to learn key properties of the known DCM-linked genes and extrapolates to identify additional such genes. Specifically, Hridaya is a supervised machine learning model to identify potential new functional genes of DCM in humans, using many different kinds of features, by learning from a gold-standard set of known functional genes. This contrasts with the previous approaches which are usually based only on differential gene expression^4–8^ or PPI networks^20^.

Several lines of evidence, including mouse knockout effects, drug side effects, and associations between regulatory variants and cardiomyopathy, support the functional role of the predicted Hridaya-genes. Many of the predicted DCM functional genes were recently shown experimentally to be mechanistically linked to cardiac diseases; notably, the TTL gene, which was very recently shown to be directly involved in microtubule buckling during cardiac contraction^34^. Hridaya predictions, along with cell line experiments, reveal important drug targets for DCM. Hridaya can be used to predict drugs likely to cause cardiac side effects and for prioritizing new drug targets for cardiomyopathy. Further, it can be used to identify approved drugs that can be repurposed for cardiac disease treatments. Specifically, drugs that are approved for non-cardiac therapies, target high-ranking Hridaya-genes, and are upregulated in DCM patients should be considered top targets for cardiac-drug repurposing. Hridaya can also predict genes having significantly different exon usage in DCM patient heart. Stratifying DCM patients, using either the expression or the genetic regulators of predicted functional genes, reveal two distinct subgroups of patients with different clinical phenotypes. Additional follow-up experiments need to be done to establish the causal role of the predicted functional genes.

Most earlier attempts that characterize important genes in cardiac diseases rely on differential gene expression^4–8^ and use only a small number of heart samples. Due to various confounders, especially co-expression among genes, the clear majority of differential genes are likely to represent downstream effects. For instance, we found that overall 54% of genes are differentially expressed between DCM and normal individuals (Wilcoxon rank sum test, p-value < 0.05); 30% are down regulated in DCM while 24% of genes are upregulated. In comparison, among the top 1000 Hridaya-genes 84% are differentially expressed. Interestingly, however, the clear majority of these genes (76%) are down regulated in DCM individuals. We also see that the top predicted functional genes are highly expressed in the left ventricle of the heart based on the RNA-seq data from GTEx consortium (see Supplementary note, Supplementary Fig. S5).

Some previous studies use animal models to identify functional genes in humans. Though important, animal models have been found in many cases to have poor translatability^47^. The previous studies did not investigate the genetic signals underlying gene expression to detect functional genes. In contrast, our approach integrates a wide range of genetic, epigenetic, transcriptomic, phenotypic, and evolutionary evidence and utilize data from 213 human hearts to predict functional genes of DCM. Such studies are important especially since it has been observed that using genetic data to select the most appropriate drug targets and indications can greatly improve the success in developing novel drugs^48^.

Hridaya’s first step, namely, the disease-functional estimator, estimates the likelihood of a gene to be a functional for some disease, i.e., not specific to DCM. Several previous works have directly addressed this broad problem, both regarding identifying functional genes as well as associated SNPs underlying diseases. As an example, a framework called Combined Annotation Dependent Depletion (CADD) uses SVM to estimate relative pathogenicity of human SNPs^49^. Polyphen-2 predicts damaging effects of missense mutations^50^. Another method called GWAVA^51^ supports the prioritization of non-coding variants by using multiple epigenomic and genomic annotations. A Bayesian approach to detect potentially causal eQTL SNPs was proposed in Das *et al*.^52^. A large-scale exome sequencing study of 60,706 individuals predicted 3230 possible disease-causing genes^53^. Network-based approaches was also used to predict disease-causing genes based on a protein-protein interaction network^20,54^. NetWAS identifies disease-gene associations using tissue-specific associations, and GWAS studies^55^. Hridaya’s first step thus provides an alternative unbiased machine-learning strategy to identify functional genes of any disease. Importantly, Hridaya integrates an additional model that discriminates among the disease-functional genes to select those specifically linked to DCM.

**Figure 5:**
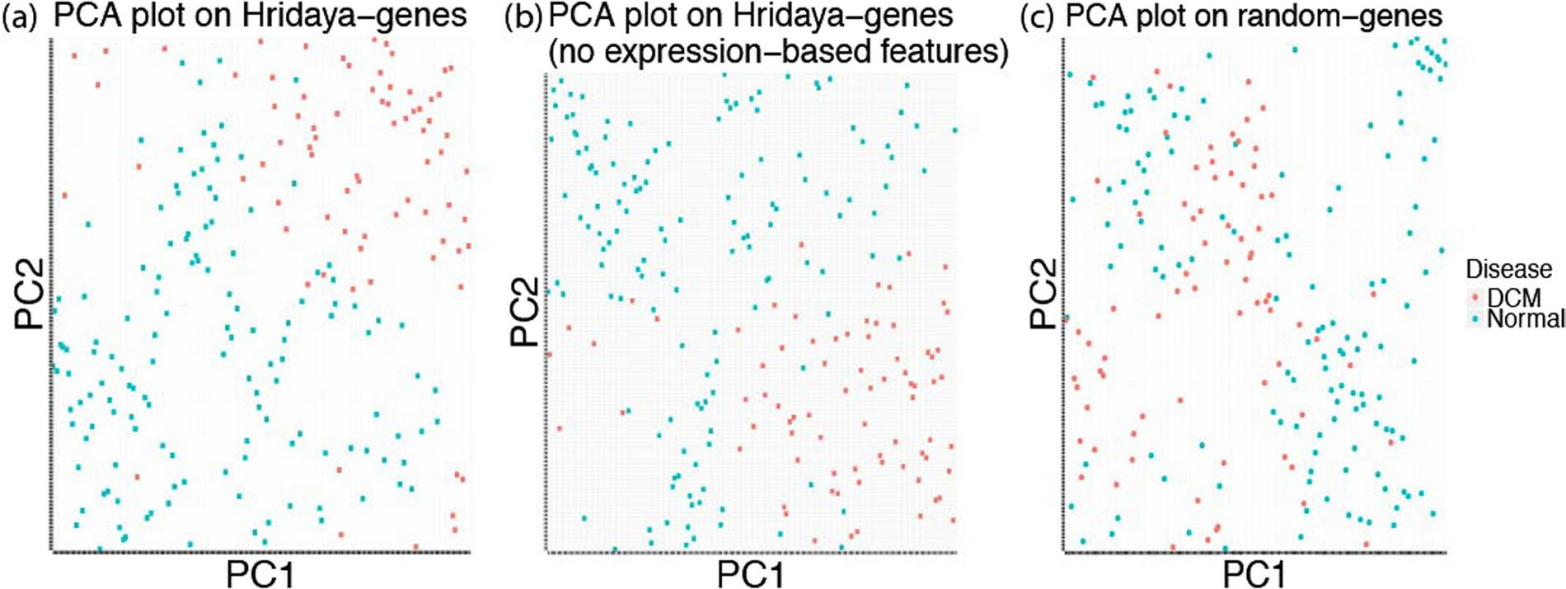
PCA plots on gene expression of (**a**) top 1000 Hridaya genes (functional genes); (**a**) top 1000 Hridaya genes (without using gene expression based features); (**c**) random 1000 genes.

We did a Principal Component Analysis (PCA) using the gene expression for the top 1000 predicted functional genes. Using only the first two principal components, we see that DCM and normal patients seem to be reasonably well separated (Fig. 5a). We also repeated this analysis by re-estimating functional genes by training Hridaya without using any gene expression based features to avoid any circularity (since we use gene expression for the PCA analysis). We again see DCM and normal patients well separated using the first two principal components (Fig. 5b). However, we do not get such a separation using random 1000 genes (Fig. 5c). This seems to suggest that expression profile of multiple functional genes is linked to DCM.

Regrettably, the DCM cohort in the MAGNet data has not been screened for known monogenic causes of DCM, which are known to contribute significantly to DCM^56–59^. Whole genome or exome sequencing data, which is necessary to address this, is currently not available. One limitation of Hridaya is its reliance on a high-quality gold-standard set of genes. Furthermore, if the gold-standard gene set is not sufficiently large, it limits the number of features that can be used, as the number of features should not be much larger than the gene set for robust model training. Although our integrative approach identified functional genes of DCM, it does not clarify the precise mechanisms by which the functional genes affect DCM. However, a detailed list of high-confidence potential DCM functional genes should serve as useful resource to pursue more directed experimental approaches to probe the mechanisms underlying DCM.

Overall, we present a novel approach to identify genes underlying DCM, supported by multiple lines of evidence, and provide a resource for future clinical investigations of DCM. Our approach is generalizable to other complex diseases. While our method predicts putative functional genes involved in DCM, establishing causality and the mechanisms will require direct experimental work in animal models. Our work provides promising candidates for such experimental follow up.

## Methods

### Hridaya method

From the Human Phenotype Ontology (HPO) database, we collect a gold-standard set of 214 positive genes that are associated with phenotypes related to DCM. Our negative control set is all the genes available in MAGNet database minus the positive set (26,590 genes). Hridaya hierarchically estimates a potential of a gene to be DCM functional genes in a supervised fashion. Hridaya uses two supervised models using support vector machines (SVMs). The SVMs are based on 181 features broadly grouped into (1) genetic, (2) epigenetic, (3) transcriptomic, (4) phenotypic, and (5) evolutionary. The list of 181 features is provided in the Supplementary Table S1 along with the details on how they were processed.

For the first SVM model, called disease-functional estimator, is based on a positive set of 3373 disease-associated genes in the HPO dataset, and the rest of the genes as the negative set. We train a SVM model using the two sets of genes to estimate the probability of each gene belonging to the positive set – P’(Disease-functional|All) and iterate this procedure 100 times (we randomly sample the much larger negative set each time so as to make it of equal size as the positive set). Multiple previous studies have shown that it is necessary to use balanced positive and negative datasets for improved performance^60–62^, thereby justifying the need for sampling methods for imbalanced datasets. Random subsampling of the negative set when it is much larger than the positive set is a common machine learning approach in classification problems^60,63^. Therefore, we did random subsampling of our negative set since our negative set is much larger than the positive set.

We then compute the average probability score for each gene (trimmed mean by removing the highest and lowest 5%). For the second SVM model, called DCM-component estimator, the positive set is comprised of genes in the HPO dataset related only to DCM (214 genes) while the negative set is 3159 (3373-214) genes associated with any disease except DCM (with random sampling as before). We then predict the probability of each gene belonging to the positive set – P’(DCM|Disease-functional). As before, we iterate this procedure 100 times and compute the trimmed average probability score. For a given gene, the product of the two probabilities P’(DCM|All) = P’(Disease-functional|All)xP’(DCM|Disease-functional), called Hridaya-potential, is the final estimated potential of a gene to be a DCM functional gene. The term ‘probability’ denotes the likelihood that a gene is functionally involved in DCM, the higher the value the more likely it is to be a functional gene of DCM.

Representing the overall potential of a gene to be DCM functional gene as a product of two probabilities, i.e., P’(DCM|All) = P’(Disease-functional|All) × P’(DCM|Disease-functional), derives from a simple probabilistic reasoning as follows. As the set of disease-functional genes is a subset of all genes P’(DCM|Disease-functional) = P’(DCM|Disease-functional, All). Now, P’(DCM|Disease-functional, All) × P’(Disease-functional|All) = P’(DCM,Disease-functional|All). Furthermore, as the set of DCM functional genes is a subset of disease functional genes P’(DCM,Disease-functional|All) = P’(DCM|All). Hence, P’(DCM|All) = P’(Disease-functional|All) × P’(DCM|Disease-functional).

Finally, our MAGNet expression data is based on Affymetrix microarray. We estimate the probabilities for each probe separately and take the highest potential among the probes mapping to a gene as the gene-level Hridaya-potential.

### Data processing

As detailed in a previous publication^52^, 313 cardiac tissue samples were acquired by the MAGNet database (http://www.med.upenn.edu/magnet/). Out of this 77 samples are from DCM human hearts and 136 from donor controls. Samples were taken from the left ventricular free-wall tissue and were harvested during cardiac surgery from heart failure patients undergoing transplantation and from unused donor hearts^52^. An empirical Bayes method called ComBat, was used to remove potential batch effects in gene expression values^64^. More details on data processing are provided in Das *et al*.^52^

The ENCODE^11^ and Roadmap epigenome project data^12^ were used for epigenetic and DNase hypersensitivity data. The data was processed as in Das *et al*.^52^. For the phylogenetic profile features based on 65 species, orthology relationships between human and each of the other species were obtained from Ensemble Biomart and then merged into one table by human gene id. More details on how the various features were processed are given in Supplementary Table S1. Missing data in the features are imputed using imputePCA function in R.

### Cross-validation and comparative methods

We did five-fold cross validation (using DCM-linked genes from HPO dataset and the rest of the genes as control) to estimate the model accuracy. 50 randomized iterations were done. For each iteration, there were 10 iterations done for each of the two components, disease-functional estimator and DCM-component estimator.

#### Conventional differential gene expression predictor

To compare with the standard gene prioritization approaches based on differential expression, we ranked each gene based on the negative log p-value of its differential expression (Wilcoxon rank sum test) between DCM and normal individuals.

#### Cipher

We also compared Hridaya with another method called Cipher^20^. Cipher predicts and prioritizes disease-genes for several phenotypes using human protein-protein interactions. We used the Cipher predictions for dilated cardiomyopathy based on the extended protein-protein interaction network they used, which gives a ranked list and disease associated score for all genes^20^.

#### Predictive value of individual Hridaya features

ROC-AUC values using one feature at a time is also computed, by using 10 randomized iterations for each feature (Supplementary Table S1). Furthermore, to estimate the predictive power of a feature separately in each of the two Hridaya components (Disease-functional estimator and DCM-component estimator), we compared the feature values between the positive and the negative set using Wilcoxon rank-sum test.

### Validations

For validations using mouse knockout data, 34 genes were compiled from the mouse knockout database^22^, whose knockout resulted in phenotypes which were associated with DCM. We removed the genes which are a part of known DCM-linked genes (gold-standard set) while using Hridaya. We computed the ROC-AUC by using this set as the positive set and the rest of the genes as the negative set, using the corresponding Hridaya-potentials for each gene. To estimate confidence interval for ROC-AUC, we bootstrap (by random sampling with replacement) the positive set (corresponding negative set is simply the complement), and repeated the procedure 100 times. ROC-AUC for each bootstrap iteration is computed, and these values for 100 bootstrap iteration are compared with those for a competing method (using Wilcoxon test) to estimate the p-value, or used to calculate confidence intervals. We also repeated the bootstrapping using pROC R package^65^ to compute confidence intervals. When we compare our approach with competing methods using these bootstrapped values, we get extremely significant p-values (<2.2e-16) using Wilcoxon test.

A similar procedure was carried out for the other validations like cardiac side effect drug targets, cardiac drugs, and genes with significant differential exon usage between DCM and normal patients. Again, known DCM-linked genes (gold-standard set) from the HPO dataset was removed from the positive (drug targets or genes with differential exon usage) and negative sets (remaining genes) for each of this analysis, so as to avoid any bias.

### Cell-line experiments

We downloaded Level 1 Data (Unique Molecular Identifier Counts) from the Drug Toxicity Signature (DToxS) Generation Center (martip03.u.hpc.mssm.edu/data.php) from the most recent three releases: August 20th, 2015, March 1st, 2016, and January 5th, 2017. We downloaded the Level 2 Data for the individual experiments from these releases to obtain the control and experimental sample names. The data contains 4 cell lines derived from cardiomyocytes from healthy hearts, 7 drugs which are known to be cardiotoxic (offending drugs), and 9 mitigating drugs which reduces the adverse effect of the offending drugs. In total, we have 28 cell-line drug pair combinations. Using the raw RNA-seq counts (Level 1 Data), we identified genes that were differentially expressed (with FDR < 0.1) between the control and post-treatment samples using the R package edgeR^66,67^. We selected genes that were differentially expressed after treatment with the offending drug in a given cell line in the same direction as in the idiopathic dilated cardiomyopathy from the MAGNet data set but were either not differentially expressed or differentially expressed in the opposite direction after treatment with the same offending drug and a mitigating drug in the same cell line. We counted the number of times each gene was selected across the 28 cases where both sets of experiments were performed and selected the 2349 genes that occurred at least in two cell line drug pair combinations. Next, we removed all known DCM-linked genes and compared the Hridaya score for the positive set of genes with those for the rest of genes present in DToxS data as well as considered by Hridaya, using the Wilcoxon Rank Sum test. Finally, we ranked all the genes (excluding the known DCM-linked genes) by their Hridaya-potential and the negative log (p-value) (conventional differential expression predictor) of their differential expression and compared the ranks of the positive set based on the two measures using the Wilcoxon Rank Sum test.

### Gene-gene interaction network

To check if differentially expressed genes are closer to predicted functional gene than expected in the gene-gene interaction network, we considered the top and bottom 100 Hridaya ranked genes present in the human functional gene-gene interaction network^37^. We also selected all the differentially expressed genes (4751 genes) between normal and DCM individuals (Wilcoxon p-value < 0.05) present in the gene-gene interaction database (after removing the overlapping top and bottom 100 Hridaya ranked genes). For each differentially expressed gene, we computed its shortest path to each of the top 100 Hridaya-genes and noted the mean and minimum values across the 100 values. As a control, we estimate the same relative to bottom 100 Hridaya-genes. Wilcoxon rank-sum test was used to find the difference between the shortest path lengths (separately for mean and minimum shortest paths) from differential genes to top ranked genes and differential genes to bottom ranked genes. This was repeated for different network stringencies (see Supplementary Fig. S2).

To remove any bias due to higher co-expression of differential genes with the predicted functional genes, we did the following analysis. We selected the top 100 functional genes which are present in the gene-gene interaction network and for each gene compute its expression Spearman correlation with the genes that are differentially expressed between DCM and normal (foreground). From the negative set of non-differentially expressed genes (remaining genes), we randomly sample 1000 genes such their expression correlation values with respect to functional genes have same distribution as that for the differentially expressed genes. We compute the shortest path of predicted functional genes with respect to the foreground (differentially expressed genes) and selected background, controlling for the co-expression with the functional genes.

Next, we assessed whether the known DCM-linked genes are closer to the top Hridaya-genes than the rest of the genes. To check this, using the interaction network, we computed the distances between known DCM-linked genes to the top novel Hridaya-genes. We also computed the distance between the known DCM-linked genes and the rest of the genes. A one-sided Wilcoxon rank-sum test was done to see if known DCM-linked genes are closer to the top Hridaya-genes than the rest of the genes (see Supplementary note).

### Predicting DCM risk based on SNPs associated with functional gene expression

We obtained the eSNPs (FDR < 0.1) for the top 1000 Hridaya-genes using matrix eQTL^38^. For this the functional genes were re-predicted after removing features related to eSNPs, so as to avoid any circularity. Then for each individual, for each functional gene, we obtained the mean value of the genotypes across all eSNPs of that gene whose FDR < 0.1. Thus, each gene corresponds to a single feature and an individual is represented by 1000 features corresponding to 1000 top functional genes. We then trained an SVM to classify DCM versus healthy donor hearts in MAGNet. Randomized 5-fold cross validation was done 100 times.

### Web-portal

We have set up a web-application where one can search for genes and the Hridaya-potential, rank, and details of these genes, at https://nishanthnair.shinyapps.io/heartdisease.

## Electronic supplementary material

Supplementary note

Supplementary table S1

Supplementary table S2

Supplementary table S3

Supplementary table S4

Supplementary table S5
