# Supplementary material for: Putative functional genes in idiopathic dilated cardiomyopathy
Source: Sci Rep. 2018 Jan 8;8:66. doi: 10.1038/s41598-017-18524-2 (PMC5758757; doi:10.1038/s41598-017-18524-2)
Supplement: Supplementary file 1 — Supplementary note [file 41598_2017_18524_MOESM1_ESM.doc]

Supplementary Note for paper “Putative Functional Genes in Idiopathic Dilated Cardiomyopathy”

*Nishanth Ulhas Nair^1,#,*^, Avinash Das^1,#^, Uri Amit^2,3,4,5^, Welles Robinson^1^, Seung Gu Park^1^, Mahashweta Basu^1^, Alex Lugo^1^, Jonathan Leor^2,3^, Eytan Ruppin^1,6^, and Sridhar Hannenhalli^1,*^*

# equal contribution
* corresponding authors ([nnair@umiacs.umd.edu](mailto:nnair@umiacs.umd.edu), [sridhar@umiacs.umd.edu](mailto:sridhar@umiacs.umd.edu))
1 – Center for Bioinformatics and Computational Biology, University of Maryland, College Park, Maryland 20742, USA.
2 – Neufeld Cardiac Research Institute, Tel Aviv University, Israel.
3 – Tamman Cardiovascular Research Institute, Sheba Medical Center, Israel.
4 – The Dr. Pinchas Borenstein Talpiot Medical Leadership Program, Sheba Medical Center, Tel-Hashomer, Israel.
5 – Department of Radiation Oncology, Sheba Medical Center, Tel-Hashomer, Israel.
6 – The Blavatnik School of Computer Science, Tel Aviv University, Tel Aviv 69978, Israel.


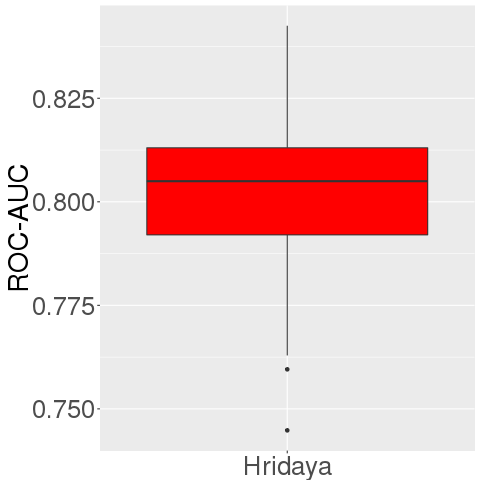


***Figure S1.*** *Box plot of five-fold cross validation results for Hridaya run over 50 iterations.*


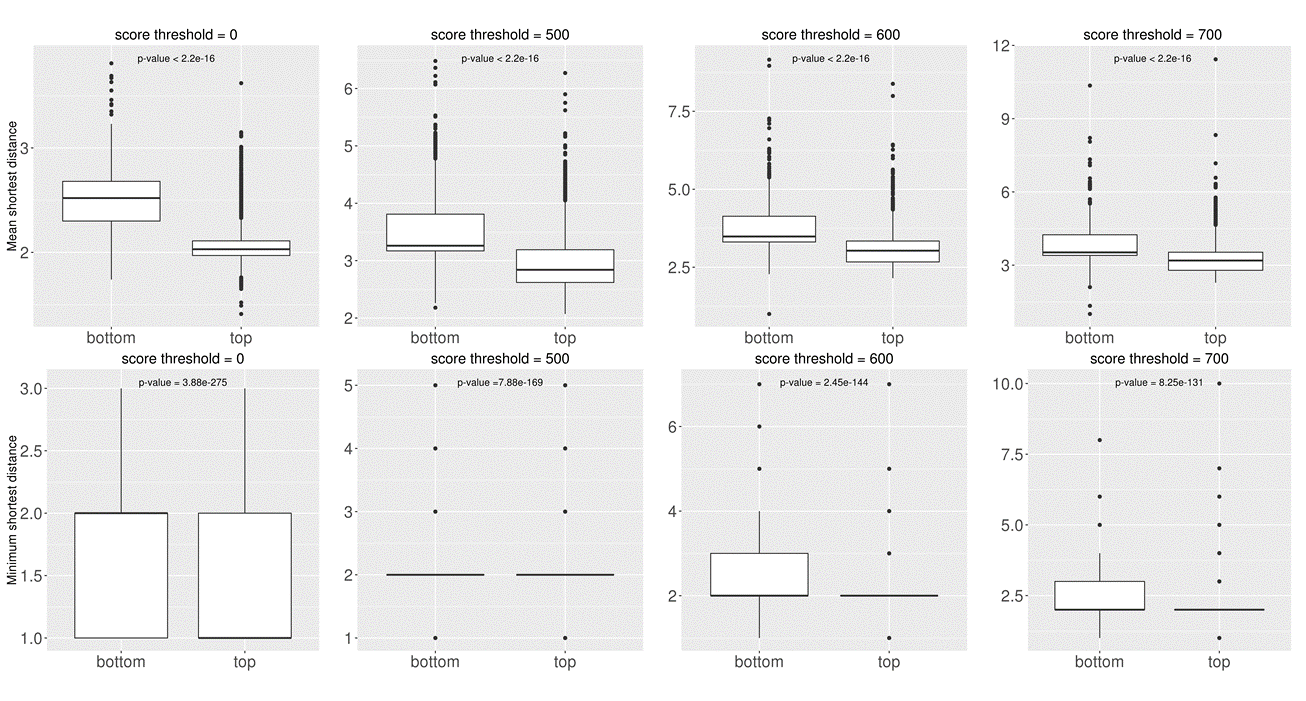


***Figure S2:*** ***Shortest path between Hridaya-genes and differentially expressed genes.*** *The top and bottom 100 ranked Hridaya predicted genes present in interaction database are selected. The shortest paths to these genes from each differentially expressed gene are calculated. Top row: box plot containing the mean shortest path length of each differential gene to a Hridaya-gene. Bottom row: box plot containing the minimum shortest path length of each differential gene Hridaya-gene. Each column shows the experiment repeated for different values of score thresholds of STRING database.*


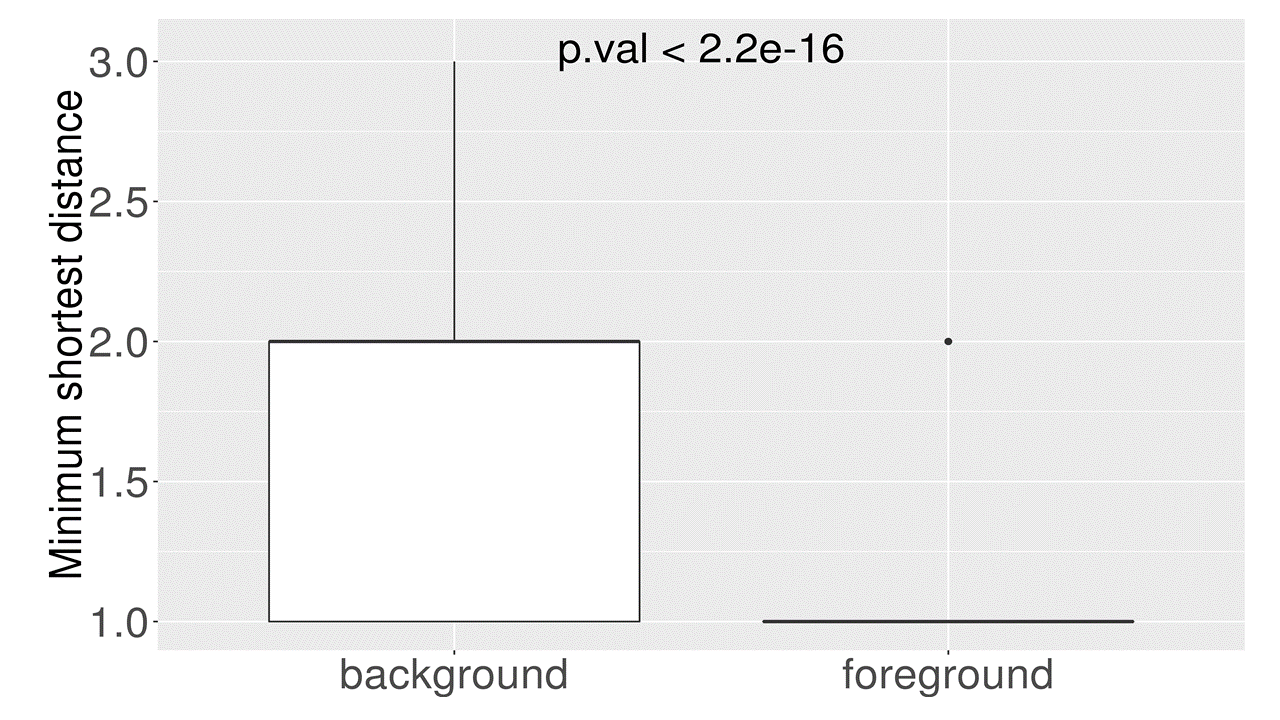


***Figure S3:*** *Minimum shortest paths of the predicted functional genes from differentially expressed genes (foreground) and randomly sampled non-differential genes (background) which have the same co-expression distribution (computed with respect to functional genes) as the foreground. We see that the predicted functional genes are closer to foreground genes than random background (one sided Wilcoxon test p-values are also shown).*

*
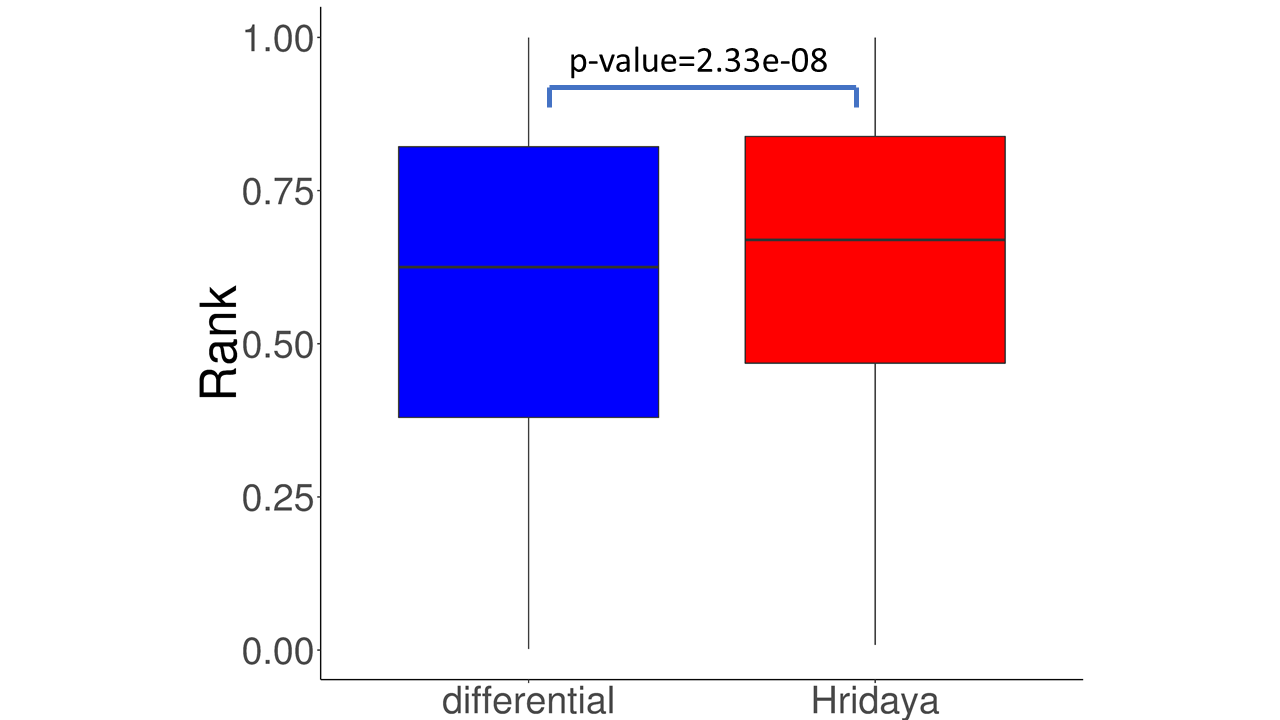
*

***Figure S4.*** *The figure shows the rank distributions of selected genes from cell line experiments (DToxS data) using Hridaya rank and the conventional differential gene expression rank. We see that Hridaya has a higher rank (with p-value=2.33e-08). For the sake of visualization, ranks are normalized from 0 to 1, with 1 being the top most rank.*


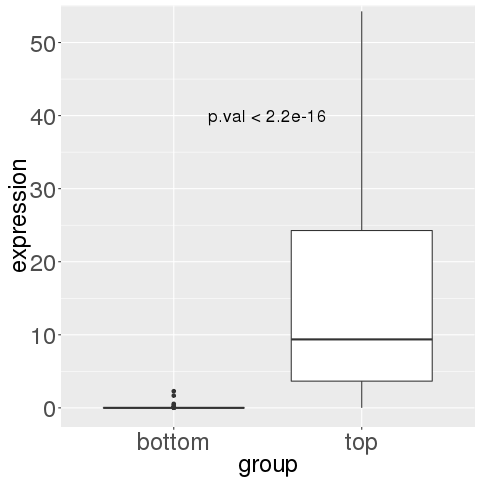


***Figure S5.*** *RNA-seq expression values of Left Ventricle (from GTEx consortium data) of top and bottom 1000 predicted functional genes (using Hridaya).*

- The word Hridaya means Heart in Sanskrit. Hridaya can be pronounced as HRI-DA-YA.

**Known DCM-linked genes are closer to the top Hridaya-genes than the rest of the genes.**

To check if the known DCM-linked genes are closer to the top Hridaya-genes than to the rest of the genes, we used the human functional gene-gene interaction network^1^ as a proxy for regulatory network. The distances between known DCM genes to the remaining Hridaya-genes (top 1000 predicted functional genes after removing the gold-standard-set of known DCM genes) was computed and also the distance between the known DCM genes and the rest of the genes (which are not known DCM genes or Hridaya-genes) were computed. A one-sided Wilcoxon rank-sum test showed that known DCM functional genes are much closer to the remaining Hridaya genes than to the rest of the genes. This was repeated for different network stringencies, we get p-value<2.2e-16 for all score thresholds.

**Discussion of few top Hridaya-genes previously not associated with DCM**

We took a closer look at Hridaya-genes hitherto not linked to cardiomyopathies. We discuss a few cases here and provide the rest of the genes in Supplementary Table S2(a). For instance, NID1 is associated with calcium ion and laminin binding, and calcium ions need to be tightly regulated for cardiac muscle relaxation and contraction^2^. MRAS is a protein coding gene (member of Ras family) involved in cell differentiation and cell growth; with GO terms including GTP binding and GTP-dependent protein binding^3^. MRAS gene is suggested to be associated with atherothrombotic stroke and coronary artery disease^4^. AK1 is involved in metabolism pathways and has kinase activity, and the creatine kinase system and protein kinases are associated with cardiomyopathy^5^. LDHD exhibits oxidoreductase activity and related enzymes such as Xanthine oxidoreductase have been associated with heart failure^6^. FOXJ2 is a transcription factor associated with heart development^7^. RERE gene is associated with diseases such aortitis, which is an inflammation of the aortic valve^3^.

**Top Hridaya genes tend to be highly expressed in heart in independent RNA-seq dataset**

We obtained the RNA-seq data from the Left Ventricle from GTEx consortium. We compared the top 1000 with the bottom 1000 Hridaya-ranked genes. We find that the top 1000 predicted functional genes have much higher heart expression than the bottom 1000 (one sided Wilcoxon test, p-value < 2.2e-16, ratio of median expression of top 1000 and bottom 1000 Hridaya ranked genes is 1.7e+08, Fig. S5).

**Functional enrichment of predicted functional genes reveals many cardiac functions**

We performed a functional enrichment analysis of the top 250 Hridaya-genes (excluding the known DCM genes used for training) based on KEGG pathways and GO biological processes. These analyses reveal association with many cardiac functions (Supplementary Table S4). Notably, KEGG analyses revealed Cardiac muscle contraction (FDR=4.34e-03), Hypertrophic cardiomyopathy (FDR=1.76e-02), Dilated cardiomyopathy (FDR=2.16e-02), etc. Consistently, the GO analyses revealed Regulation of heart rate (FDR = 4.44e-02), Regulation of striated muscle contraction (FDR=2.91e-08), Muscle organ development (FDR=2.94e-2), Cardiac conduction (FDR=2.29e-04), Cardiac muscle cell action potential (FDR=2.05e-03), etc. These functional enrichments unequivocally point toward the cardiac function of these genes, despite the fact that functional annotations were not used in the model.

**Precision-recall calculation**

Our goal is to train a model based on well-established gold set of 214 genes and then to identify novel functional genes from the rest of the genes. That is, we expect several positives in our ‘negative’ set. This can drastically and adversely affect precision-recall metric. For instance, in an idealized situation, is there are 1000 unknown functional genes in the negative set and we identify them perfectly, even then, the precision will be estimated as True Positive / (True Positive + False Positive) = 214/(214 + 1000) = ~18%. In other words, our idealized perfect classifier will seem as performing poorly according to precision value. Thus, precision-recall (unlike ROC) is highly sensitive to hidden positives in the negative set, and hence is not an ideal measure for our purpose.

So, to compute precision-recall in a reasonably accurate and unbiased manner, for validation, we filter out genes from the negative set which may contain true positives. To do this, in the validation set, we only included a gene in the negative set if (a) the gene was not differentially expressed between DCM and normal individuals (in heart tissue), and (b) the gene was lowly expressed in heart (bottom 50th percentile). We did this filtering of the negative set based on the premise that genes that have low heart expression and that are not differentially expressed in disease-versus-normal hearts are unlikely to be a functional gene. We then train Hridaya as before using all genes, but while testing we use the new universe of positive (214 genes) and negative sets (9504 genes compared with 26590 before). Doing so, we obtain a precision-recall AUC of 0.473 (based on 5-fold cross validation). However, to ensure that our results are not biased by gene expression (because we use that to filter the negative set), we repeated the analysis by excluding all expression-related features when training the model (this resulted in 115 features as opposed to 181 originally). With the new reduced feature space, we still get a good precision-recall value of 0.245 (median value across 20 iterations).
**Genotype data processing**

For the computation of eSNPs matrix eQTL (Shabalin 2012) was used. Data was processed as done in Das et al.^8^. Affymetrix Genome Wide SNP Array 6.0 was used to genotype DNA samples following manufactures instructions. PLINK, a software package, was used to carry out the analysis^9^. SNPs with with minor allele frequency (MAF) < 15%, genotype call rate < 95%, or if there was strong deviation from Hardy-Weinberg equilibrium (p-value < 1e-6) were eliminated^8^. We obtained 360,046 SNPs. Genotype imputation using Minimac (v2012.11.16) program^10^ was done. Filtering of imputation results was done at an imputation quality threshold of 0.5 and a MAF threshold of 0.15. We obtained a total of 6,536,277 SNPs. ComBat was used to remove potential batch effects in gene expression values^11^. PEER was used to normalize gene expression^12^. Linkage disequilibrium (LD) blocks of the genotypes were inferred using PLINK. To estimate this, the default setting of SNPs within 200 kb was used^8^. SNPs whose distance were less than 1 MB from gene transcription start site was used for eQTL computations.

**References**

1. Szklarczyk, D. *et al.* STRING v10: Protein-protein interaction networks, integrated over the tree of life. *Nucleic Acids Res.* **43,** D447–D452 (2015).

2. Pinnell, J., Turner, S. & Howell, S. Cardiac muscle physiology. *Contin. Educ. Anaesthesia, Crit. Care Pain* **7,** 85–88 (2007).

3. Safran, M. *et al.* GeneCards Version 3: the human gene integrator. *Database (Oxford).* **2010,** baq020 (2010).

4. Liu, H. *et al.* Mras genetic variation is associated with atherothrombotic stroke in the Han Chinese population. *J. Clin. Neurol.* **9,** 223–230 (2013).

5. Khuchua, Z. A. *et al.* The creatine kinase system and cardiomyopathy. *Am. J. Cardiovasc. Pathol.* **4,** 223—234 (1992).

6. Tziomalos, K. & Hare, J. M. Role of xanthine oxidoreductase in cardiac nitroso-redox imbalance. *Front. Biosci.* **14,** 237–262 (2009).

7. Tilton, R. K., Wilkens, A., Krantz, I. D. & Izumi, K. Cardiac manifestations of pallister-killian syndrome. *American Journal of Medical Genetics, Part A* (2014). doi:10.1002/ajmg.a.36413

8. Das, A. *et al.* Bayesian integration of genetics and epigenetics detects causal regulatory SNPs underlying expression variability. *Nat. Commun.* **6,** 8555 (2015).

9. Purcell, S. *et al.* PLINK: A tool set for whole-genome association and population-based linkage analyses. *Am. J. Hum. Genet.* **81,** 559–575 (2007).

10. Howie, B., Fuchsberger, C., Stephens, M., Marchini, J. & Abecasis, G. R. Fast and accurate genotype imputation in genome-wide association studies through pre-phasing. *Nat. Genet.* **44,** 955–959 (2012).

11. Johnson, W. E., Li, C. & Rabinovic, A. Adjusting batch effects in microarray expression data using empirical Bayes methods. *Biostatistics* **8,** 118–27 (2007).

12. Stegle, O., Parts, L., Piipari, M., Winn, J. & Durbin, R. Using probabilistic estimation of expression residuals (PEER) to obtain increased power and interpretability of gene expression analyses. *Nat. Protoc.* **7,** 500–7 (2012).
